# Data Provenance in Biomedical Research: Scoping Review

**DOI:** 10.2196/42289

**Published:** 2023-03-27

**Authors:** Marco Johns, Thierry Meurers, Felix N Wirth, Anna C Haber, Armin Müller, Mehmed Halilovic, Felix Balzer, Fabian Prasser

**Affiliations:** 1 Medical Informatics Group Berlin Institute of Health at Charité – Universitätsmedizin Berlin Berlin Germany; 2 Institute of Medical Informatics Charité – Universitätsmedizin Berlin Berlin Germany

**Keywords:** data provenance, biomedical research, scoping review, systematization, comparison

## Abstract

**Background:**

Data provenance refers to the origin, processing, and movement of data. Reliable and precise knowledge about data provenance has great potential to improve reproducibility as well as quality in biomedical research and, therefore, to foster good scientific practice. However, despite the increasing interest on data provenance technologies in the literature and their implementation in other disciplines, these technologies have not yet been widely adopted in biomedical research.

**Objective:**

The aim of this scoping review was to provide a structured overview of the body of knowledge on provenance methods in biomedical research by systematizing articles covering data provenance technologies developed for or used in this application area; describing and comparing the functionalities as well as the design of the provenance technologies used; and identifying gaps in the literature, which could provide opportunities for future research on technologies that could receive more widespread adoption.

**Methods:**

Following a methodological framework for scoping studies and the PRISMA-ScR (Preferred Reporting Items for Systematic Reviews and Meta-Analyses Extension for Scoping Reviews) guidelines, articles were identified by searching the PubMed, IEEE Xplore, and Web of Science databases and subsequently screened for eligibility. We included original articles covering software-based provenance management for scientific research published between 2010 and 2021. A set of data items was defined along the following five axes: publication metadata, application scope, provenance aspects covered, data representation, and functionalities. The data items were extracted from the articles, stored in a charting spreadsheet, and summarized in tables and figures.

**Results:**

We identified 44 original articles published between 2010 and 2021. We found that the solutions described were heterogeneous along all axes. We also identified relationships among motivations for the use of provenance information, feature sets (capture, storage, retrieval, visualization, and analysis), and implementation details such as the data models and technologies used. The important gap that we identified is that only a few publications address the analysis of provenance data or use established provenance standards, such as PROV.

**Conclusions:**

The heterogeneity of provenance methods, models, and implementations found in the literature points to the lack of a unified understanding of provenance concepts for biomedical data. Providing a common framework, a biomedical reference, and benchmarking data sets could foster the development of more comprehensive provenance solutions.

## Introduction

### Background

The replication crisis has exposed a lack of reproducible results in many scientific studies, including those in the biomedical domain [1]. This phenomenon indicates that only a small fraction of published research results can be reliably and fully replicated. However, the need to improve the reproducibility of research has not only been recognized since the dawn of the replication crisis [1] but has also already received increasing attention over the past decade through initiatives such as the Findable, Accessible, Interoperable, and Reusable principles [2]. Problems with the reproducibility of research projects and their results can have many different causes and can, therefore, be mitigated in many ways. Important examples include a lack of documentation regarding experimental parameters and a lack of downstream processing of data in the form of well-defined and structured metadata, which are needed for interpretation and reproduction [3]. Both aspects are closely related to data provenance, which refers to the origin, processing, and movement of data. Reliable and precise knowledge about data provenance has great potential to assess and improve reproducibility as well as quality in biomedical research and, therefore, to foster good scientific practice [4,5].

Although the definitions of data provenance information vary in some aspects, it is generally understood as metadata, which describe all events that influenced a data set. A data set can be altered by some processes, resulting in a changed state. We consider a data set with a changed state to be a new data set. Data provenance tracks information about its conception (eg, who or what created the data) and all transformations and processing operations that may have been applied [6]. This can be used to identify potentially invalid processing steps, data quality degradation, or limitations for secondary use [3,4,6]. Terms such as data lineage and data pedigree can have slightly different meanings in some of the literature (eg, pedigree is sometimes understood as also capturing information about the quality or trustworthiness of data sources [3,4]) but are often also used interchangeably with provenance (eg, the studies by Simmhan et al [6] and Baum et al [7]), which is the approach we follow in this paper.

In the biomedical context, data are collected in many forms and types as well as for different purposes, including health care and research. Usually, such data include information about treatments, conditions, and outcomes of a patient, which are often described by measurements or more abstract observations. The origin of such observations and the context in which they have been collected can differ, which can have consequences for their meaning and reliability. For example, observations can be manually captured by a person (eg, a health care professional measuring the heart rate of a patient) or automatically captured by a device (eg, a digital pulse oximeter already placed on the patient’s finger), influencing their precision. Another example would be deriving structured research data from clinical documents, which can be a manual process involving curation or an automated process performed by machines, which impacts reliability. Considering the previously mentioned processing of such data and errors or inaccuracies potentially introduced along the way, the assessment of data provenance metadata (eg, by visualization or analysis) can help clinicians or researchers understand the quality of information and informaticians find the root causes in case of problems.

**Figure 1:**
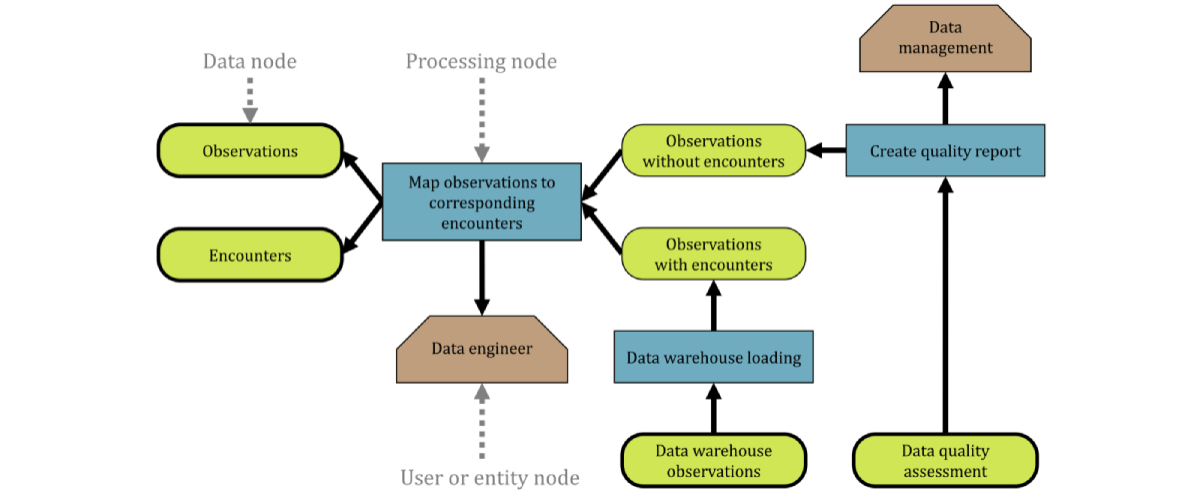
A simple example provenance graph, where observations are mapped to encounters to be loaded into a data warehouse.

Figure 1 shows an example of a provenance graph based on commonly used provenance data models, such as PROV [8,9] and the Open Provenance Model (OPM) [10], which consists of data nodes, processing nodes, and user or entity nodes (sometimes also called agent nodes), which are linked by directed edges, representing the relationships among the nodes (eg, the processing node responsible for the creation of the respective data or denoting responsible entities).

In this graph, the input data nodes represent data on *observations* and *encounters*, for example, from an eHealth record system. In the first processing step, the observations are *mapped to corresponding encounters* before loading them into a data warehouse. This falls under the responsibility of a *data engineer*. The observations that cannot be assigned to an encounter are processed to *create a quality report*, which is overseen by the * data management* entity, resulting in the *data quality assessment* data node. The observations with an encounter are loaded into the data warehouse, resulting in the *data warehouse observations* data node. Having complete and plausible data, for example, as indicated in such a data quality assessment, is of importance in research. This is true not only for data coming from carefully planned studies but also for data coming from other contexts (eg, health care data used for secondary purposes), as they can contain unexpected issues requiring data inspection and cleaning [11]. Moreover, data are increasingly gathered in an automated manner by sensors and other devices, where provenance should be accurately reported to provide a wholistic picture of the conception of the data and all the factors regarding their quality and fitness for use [12].

Data provenance can be captured prospectively and retrospectively, relative to when data processing occurs [3,13,14]. Prospective generation has the advantage that the provenance capturing methods can be integrated directly into data generation, transformation, and analysis pipelines to automatically and accurately gather the complete information of such processes in necessary detail. Retrospectively, it might still be possible to obtain some provenance information, but this usually comes with limitations in what details can be included [14]. For instance, provenance metadata can be retrospectively derived from log files, which may not contain all the information about every processing step or contain information with insufficient details, as log files are often meant to be human readable for troubleshooting.

### Objective

Although data provenance tracking is a common practice in some disciplines, such as physics, geoscience, geography (particularly in geographic information systems), material science, hydrologic science, and environmental modeling [15-19], it has yet to be widely adopted in many other data-driven research disciplines, including biomedical research [7]. Consequently, previous reviews either focused on provenance outside the biomedical context (eg, the studies by Simmhan et al [6] and Herschel et al [3]) or studied a larger spectrum of data generation and preparation activities of which provenance is just one aspect (eg, the study by de Lusignan et al [4]). This raises the question of whether the methods suggested to date have weaknesses or lack important functionalities that impede their use in biomedical research. To bridge this gap, we believe it is important to study the literature focusing solely on provenance management methods developed for or used in the biomedical domain and the similarities and differences between them (refer to the *Related Work* section for a more detailed discussion).

In this paper, we present a scoping review to (1) provide a detailed overview of research describing data provenance technologies (eg, for imaging data, health records, and omics data) developed for or used in biomedical research; (2) describe and compare the supported functionalities (eg, creating, storing, querying, analyzing, or visualizing data provenance information) as well as the design of the methods (eg, use of standards or types of data storage); and (3) use this information to identify gaps in the literature (eg, combinations of functionalities that are rarely supported), which could provide opportunities for future research on technologies that could receive more widespread adoption.

## Methods

### Research Methodology

This systematic scoping review was performed in conformance with the methodological framework developed by Arksey and O’Malley [20] and reported using the PRISMA-ScR (Preferred Reporting Items for Systematic Reviews and Meta-Analyses Extension for Scoping Reviews) guidelines [21]. No ethics approval was sought, as this study analyzed data from previous studies. A protocol for this review was not published because the International Prospective Register of Systematic Reviews does not include scoping reviews [22]. Furthermore, this review does not yield or report on biomedical research outcomes but rather focuses on methodological and technological aspects of data provenance in the biomedical domain.

### Inclusion and Exclusion Criteria

Before defining the inclusion criteria, we conducted an unstructured literature search on data provenance and found that the body of literature included many studies from fields that were not within the scope of this review. On this basis, we set up an initial version of criteria to discriminate articles about the use of provenance methods in biomedical research from articles about the use of provenance methods in other capacities or disciplines, such as supply chains for pharmaceutical products or animal taxonomies. The description of the criteria was refined after a preliminary sample screening to mitigate the differences in interpretation among the authors.

We included articles that (1) described the use of data provenance, data lineage, or data pedigree information in biomedical research or a related scientific discipline and (2) described a software-based method (ie, articles focusing on purely manual provenance tracking were not eligible). Moreover, articles needed to be (3) original papers published in peer-reviewed journals or conference proceedings, (4) written in English, and (5) published between 2010 and 2021.

The exclusion criteria were formulated analogously. We excluded articles that (1) did not cover data provenance and instead focused on provenance in other contexts (eg, history, geology, or logistics); (2) did not focus on digital technologies, data, software, methods, or models for data provenance; (3) did not focus on biomedical or health-related research or data (eg, if the biomedical domain was only mentioned as one exemplary application area among many); and (4) did not describe the provenance of data and instead used provenance data (eg, for the tracking of products in supply chains).

### Sources and Search Strategy

Near-synonyms exist for “provenance,” such as “pedigree” or “lineage,” which hence had to be included as search terms. Furthermore, as described in the previous section, we needed to discriminate against articles not within the scope or context of biomedicine. For this purpose, we included the keywords “biomedical,” “medical,” and “health.”

We searched the Web of Science, PubMed, and IEEE Xplore databases, as the topic is at the intersection of medicine and computer science. The search strings used required article titles or abstracts to contain at least 1 keyword from each of the two topics and corresponding keywords reflecting the scope of the review:

1. The topic “Provenance” was captured by the terms (“data provenance” OR “data lineage” OR “data pedigree”)
2. The topic “Biomedicine” was captured by the terms (“medical” OR “biomedical” OR “health”)

The exact search strings used for the different databases are available in Multimedia Appendix 1. The final search was performed on February 7, 2022, using a computer within the network of Charité–Universitätsmedizin Berlin in Germany.

### Selection and Data Collection Processes

The selection process was performed using two consecutive screening steps: (1) screening of the titles and abstracts of all the resulting papers and (2) screening of the full texts of all the papers that were selected in the first step. Each article was screened by the first author and one coauthor. Disagreements were resolved by the last author. The reasons for excluding articles were also recorded and are provided in Multimedia Appendix 2*.* The data items to be collected (refer to the next section) were identified by reading the full articles, consecutively identifying patterns of similarity or dissimilarity between the information provided. Data extraction was performed by all the authors, and disagreements were resolved by the last author.

### Data Items and Analysis

We defined data items along five axes to generate insights into our research questions (RQs): (1) publication metadata, (2) application scope, (3) provenance aspects covered, (4) data representation, and (5) functionalities. An overview of the categories, individual items, and value sets is provided in Table 1. The data items were extracted from the articles, stored in a charting spreadsheet, and summarized in tables and figures. Owing to the heterogeneity and use case–specific nature of many of the methods and solutions described in the papers included, systematizing their properties into specific data elements was a considerable challenge. On the basis of an original list of data elements describing primarily the qualitative properties of the methods and solutions, an adjustment was made during the aforementioned sample screening to capture essential information in a comparable way.

As can be seen, we collected publication *metadata* to be able to study the development of interest in the topic relative to time or the locations of researchers. We further collected information on the *application scope* to investigate whether there were specific contexts in which or types of data for which provenance was studied and to gain insights into the motivation for studying provenance in general. Information on *provenance aspects* (“why,” “how,” “where,” and “who” following the terminology suggested in the study by Herschel et al [3]) was charted to better understand the specific types of information collected. Next, we compiled information on the data *representation and storage* model used, such as the abstract and concrete data model, and whether intermediate processing results were materialized. Moreover, we charted the use of the most common standards dedicated to provenance metadata, such as the OPM [10] and the World Wide Web Consortium (W3C) PROV standard [8]. Finally, we collected a range of information on the functionality of the solutions proposed, including which steps in the data life cycle [23] were targeted and how exactly the capturing, retrieval, analysis, and visualization of provenance information were realized.

**Table 1:** Data items for full-text charting.

Name		Description
**Publication metadata**		
	Year of publication	The year when the publication was published
	Author location	Countries in which the institutions of the first author and last author are located
**Application scope**		
	Application area	Whether the contribution can be applied in biomedical research or directly applied in health care practice
	Focus	Whether addressing the issue of data provenance was the primary focus of the publication or whether the provenance aspect was only mentioned indirectly or as complementary to an inherent necessity
	Motivation	The motivation behind the use of data provenance
	Types of data	The types of data for which provenance information was managed (options are structured clinical and health data, omics data, imaging data, sensor or device data, free text, and other types of data) or whether the contribution was data type agnostic (ie, generic data)
**Provenance aspects**		
	Where provenance	The contribution addresses the aspect of where the data originated from
	How provenance	The contribution addresses the aspect of how a specific result was produced (ie, the preceding processing steps)
	Who provenance	The contribution addresses the aspect of who (or which entity, such as organization, software, or device) was responsible or claimed ownership for the data or data processing
	Why provenance	The contribution addresses the aspect of why a certain result or data point was produced, which requires capturing all preceding processing steps and data sources
**Data representation and storage**		
	Abstract data model	The abstract data model used to represent provenance information; examples are graphs, lists, references, and composite objects
	Concrete data model	The concrete data model used to store provenance information; examples are blockchains, named graphs, relational models, and file-based storage
	Standard data model	Whether the data model was compatible with common provenance standards, such as PROV or the OPM^a^
	Immutability	Whether the provenance information was immutable
	Materialization	Whether the provenance information was virtual or materialized, that is, whether intermediate processing results were explicitly stored as complete data sets
**Functionality**		
	Creation and capture	How, or by what type of entity, the data provenance information was captured; we distinguished between additional capture through a stand-alone software, integrated by some middleware- or trigger-based approaches, inherently using blockchains, or extraction from external sources
	Querying and retrieval	How the provenance information was queried or retrieved; options are retrieval via API^b^ or GUI^c^, structured query, selective query, or an unstructured search query
	Analysis	Categorization of how the provenance information was analyzed, which helped identify contributions with similar feature sets; the categories are “generic” or use case agnostic (eg, descriptive statistics) and “specific” or use case dependent (eg, reasoning or error tracing)
	Visualization	Visualization type or method for identifying ways to visualize provenance information of information related to data provenance; details include whether the visualization was based on a graph or flow network to examine patterns in provenance visualization based on its native structure and whether specific tools were used for visualization
	Time of generation	The time of metadata generation; we distinguished between prospective generation, when the metadata are generated during data processing, and retrospective generation, when the data processing was done in the past and the metadata are generated based on previously generated artifacts, such as log files

^a^OPM: Open Provenance Model.

^b^API: application programming interface.

^c^GUI: graphical user interface.

## Results

### Overview

**Figure 2:**
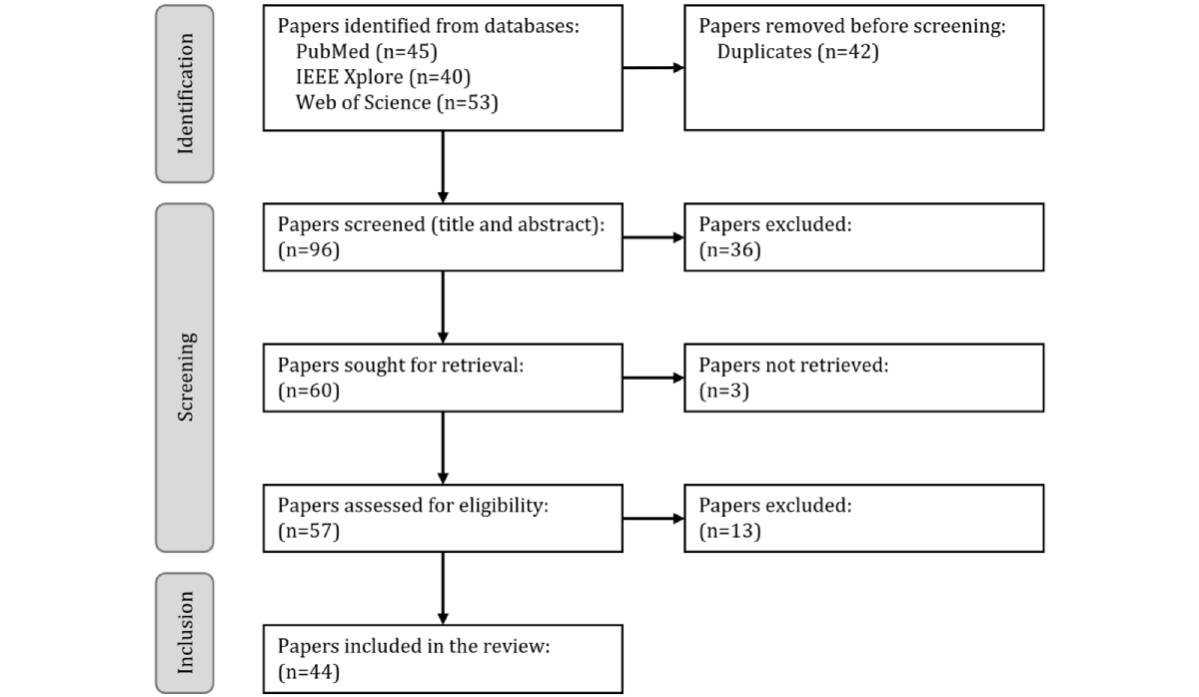
PRISMA (Preferred Reporting Items for Systematic Reviews and Meta-Analyses) flowchart for the selection process (based on the study by Page et al [24]).

A total of 138 articles were identified through the database searches (45, 32.6% from PubMed; 40, 29% from IEEE Xplore; and 53, 38.4% from Web of Science). An overview of the selection process is shown in Figure 2.

From the 138 articles, we excluded 42 (30.4%) duplicates and 36 (26.1%) articles in the first screening process. Of the 60 eligible full-text articles, 3 (5%) could not be retrieved. Of the remaining 57 articles, 13 (23%) were excluded in the second screening process. Finally, 44 articles were included in the review and processed in the data charting step (refer to Table 2 for a complete list). The resulting data items for each article are presented in Multimedia Appendix 2.

**Table 2:** List of items found eligible (n=44).

Serial number	Year	Title	Reference
1	2021	Smart Decentralization of Personal Health Records with Physician Apps and Helper Agents on Blockchain: Platform Design and Implementation Study	[25]
2	2021	Blockchain for Healthcare Data management: Opportunities, Challenges, and Future recommendations	[26]
3	2021	Adjusting For Selection Bias Due to Missing Data in Electronic Health Records-Based Research	[27]
4	2021	Risk and Compliance in IoT- Health Data Propagation: A Security-Aware Provenance based Approach	[28]
5	2021	Blockchain-Enabled Telehealth Services Using Smart Contracts	[29]
6	2021	Trellis for Efficient Data and Task Management in the VA Million Veteran Program	[30]
7	2020	A Practical Universal Consortium Blockchain Paradigm for Patient Data Portability on the Cloud Utilizing Delegated Identity Management	[31]
8	2020	Blockchain-Enabled Clinical Study Consent Management	[32]
9	2020	Decentralised Provenance for Healthcare Data	[33]
10	2020	Enhancing Traceability in Clinical Research Data Through a Metadata Framework	[34]
11	2020	Secure and Provenance Enhanced Internet of Health Things Framework: A Blockchain Managed Federated Learning Approach	[35]
12	2019	BEERE: A Web Server for Biomedical Entity Expansion, Ranking and Explorations	[36]
13	2019	Clinical Text Mining on FHIR	[37]
14	2019	Enhanced Security Framework for E-Health Systems using Blockchain	[38]
15	2019	NeuroProv: Provenance Data Visualization for Neuroimaging Analyses	[39]
16	2019	Polymorph Segmentation Representation for Medical Image Computing	[40]
17	2019	Provenance for Biomedical Ontologies With RDF and Git	[41]
18	2019	Research on Personal Health Data Provenance and Right Confirmation With Smart Contract	[42]
19	2019	The Generalized Data Model for clinical research	[43]
20	2018	Application of Data Provenance in Healthcare Analytics Software: Information Visualisation of User Activities	[44]
21	2018	Applying Blockchain Technology for Health Information Exchange and Persistent Monitoring for Clinical Trials	[45]
22	2018	BASTet: Shareable and Reproducible Analysis and Visualization of Mass Spectrometry Imaging Data via OpenMSl	[46]
23	2018	FHIR Healthcare Directories: Adopting Shared Interfaces to Achieve Interoperable Medical Device Data Integration	[47]
24	2018	ProvCaRe Semantic Provenance Knowledgebase: Evaluating Scientific Reproducibility of Research Studies	[48]
25	2018	Visualizing the Provenance of Personal Data Using Comics	[49]
26	2017	A Method of Electronic Health Data Quality Assessment: Enabling Data Provenance	[5]
27	2017	MediSyn: Uncertainty-Aware Visualization of Multiple Biomedical Datasets to Support Drug Treatment Selection	[50]
28	2017	MeDShare: Trust-Less Medical Data Sharing Among Cloud Service Providers via Blockchain	[51]
29	2017	Templates as a Method for Implementing Data provenance in Decision Support Systems	[52]
30	2016	Access Control Management With Provenance in Healthcare Environments	[53]
31	2016	Addressing Provenance Issues in Big Data Genome Wide Association Studies (GWAS)	[54]
32	2016	AVOCADO: Visualization of Workflow-Derived Data Provenance for Reproducible Biomedical Research	[55]
33	2016	Design of the MCAW Compute Service for Food Safety Bioinformatics	[56]
34	2016	TCGA Expedition: A Data Acquisition and Management System for TCGA Data	[57]
35	2015	A Platform for Leveraging Next Generation Sequencing for Routine Microbiology and Public Health Use	[58]
36	2015	Modeling Evidence-Based Medicine Applications With Provenance Data in Pathways	[59]
37	2014	Exploring Large Scale Receptor-Ligand Pairs in Molecular Docking Workflows in HPC Clouds	[60]
38	2014	Securing First-Hop Data Provenance for Bodyworn Devices Using Wireless Link Fingerprints	[61]
39	2013	Fuzzy Reasoning of Accident Provenance in Pervasive Healthcare Monitoring Systems	[62]
40	2013	Provenance Framework for mHealth	[63]
41	2013	Towards Structured Sharing of Raw and Derived Neuroimaging Data Across Existing Resources	[64]
42	2012	Improving Integrative Searching of Systems Chemical Biology Data Using Semantic Annotation	[65]
43	2012	XCEDE: An Extensible Schema for Biomedical Data	[66]
44	2011	A Provenance Approach to Trace Scientific Experiments on a Grid Infrastructure	[67]

### Publication Metadata

#### Distribution Over Time

**Figure 3:**
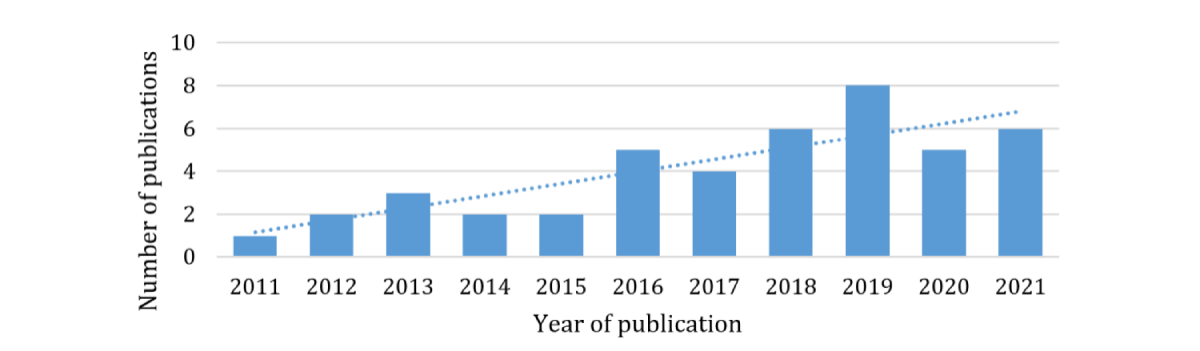
Number of publications per year.

The year of publication of the articles ranged from 2011 to 2021. Approximately two-thirds (29/44, 66%) of the articles were published from 2017 to 2021, and one-third (15/44, 34%) of the articles were published before this time frame, that is, from 2011 to 2016, pointing toward an increasing trend (Figure 3).

#### Geographical Distribution

Most first and senior authors worked at institutions located in the United States (34/90, 38%), followed by China (8/90, 9%), Germany (8/90, 9%), the United Kingdom (6/90, 7%), Australia (6/90, 7%), Canada (4/90, 4%), and the United Arab Emirates (4/90, 4%). We note that countries with fewer than 4 occurrences were pooled as “others” (20/90, 22%) and that some authors were affiliated with multiple organizations. The results are roughly comparable with the top entries in the SCImago Country Ranking [68] (categories “general” as well as “medicine”) and thus correspond approximately to the basic publication output of the respective countries.

### Application Scope

#### Application Area

Most papers (34/44, 77%) analyzed focused on provenance for research data processing only, whereas some (8/44, 18%) focused on the application of provenance in research and health care, and only 5% (2/44) specifically focused on the application of provenance in the health care practice context by presenting a backward reasoning algorithm for a monitoring system [62] or for making telehealth services transparent, immutable, and trustworthy [29].

#### Focus

In approximately half of the publications (23/44, 52%), data provenance was the primary research subject, whereas the other half (21/44, 48%) addressed data provenance indirectly or as an inherent property of a broader method or solution described.

#### Motivation

The motivations behind the need for provenance data were categorized into “validity,” “reproducibility,” “regulatory requirements,” “reusability,” and “transparency,” and each publication was assigned to the category matching the described motivation.

The most frequent reason for addressing provenance was validity (22/44, 50%), followed by reproducibility (15/44, 34%) and the need to comply with regulatory requirements (15/44, 34%), reusability (11/44, 25%), and then transparency (8/44, 18%). Some papers did not provide details on why provenance was considered (3/44, 7%). In the *Data Representation and Storage* section, we examine the relationships between the technologies used and the motivation described.

#### Types of Data Addressed

The most frequently mentioned (multiple mentions possible) supported data type was *structured clinical and health data*, such as data from eHealth records (17/44, 39%), followed by *omics* (8/44, 18%), *imaging data* (7/44, 16%), *sensor and device data* (5/44, 11%), *source references* (4/44, 9%), and *free text* (2/44, 5%). A total of 9% (4/44) of papers focused on *other* data types, including metadata or ontologies, clinical pathways, telehealth session data, and administrative data. Finally, 5% (2/44) of papers stated that the approach presented was *generic* and applicable to a wide range of data types.

**Figure 4:**
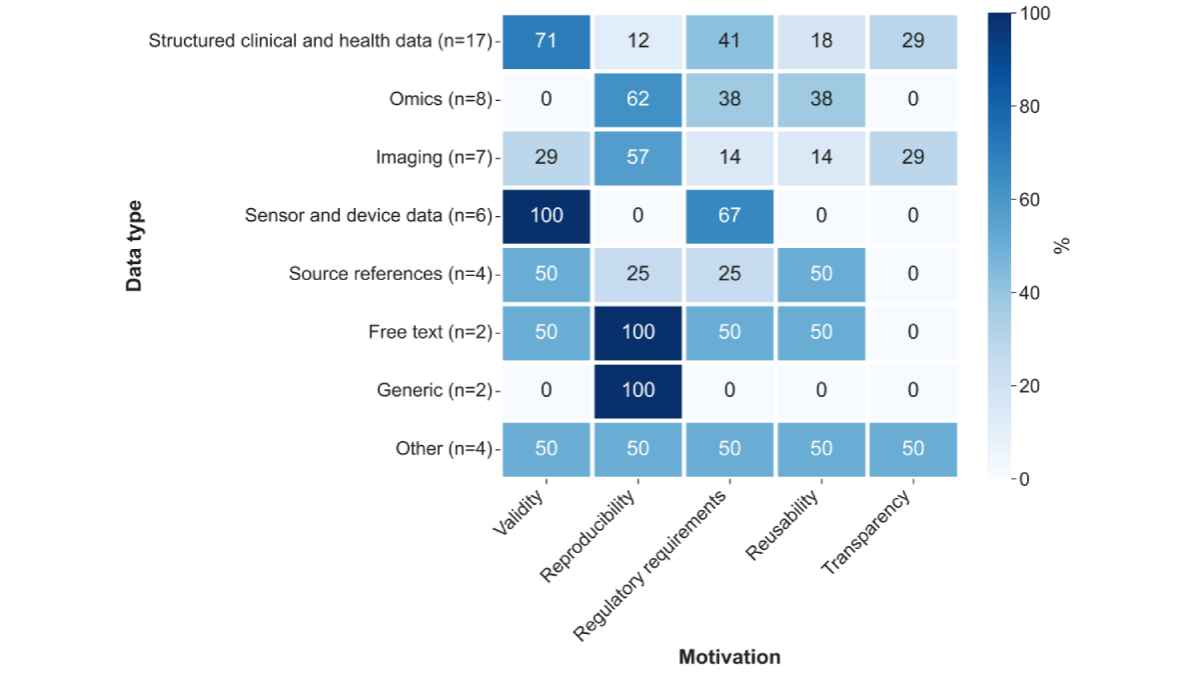
Percentage of papers addressing a certain data type and mentioning a certain motivation.

The co-occurrences of the data types focused on and the motivation presented are illustrated in Figure 4.

What stands out is that papers that addressed provenance for omics and imaging data were often motivated by reproducibility aspects. This makes sense, as both types of data are rather large and complex in nature, and processing operations, for example, bioinformatics pipelines or artificial intelligence–based image analyses, are known to sometimes be difficult to reproduce [69,70] (refer to the *Principal Findings* section for further discussion).

### Provenance Aspects

Regarding the provenance aspects supported by the methods or solutions described, we identified the coverage as provided in the following sections.

All the papers (44/44, 100%) supported *where* provenance, that is, information on where the data originated from. This is not surprising, as it can be seen as the central point behind provenance management. In addition, approximately half of the papers supported *how* provenance (25/44, 57%), that is, information on how a certain result was produced (ie, the preceding processing steps); *who* provenance (26/44, 59%), that is, information on who (or what) was responsible or claimed ownership for the data or data processing; and *why* provenance (20/44, 45%), that is, information on why a certain result or data point was produced.

### Data Representation and Storage

#### Abstract Data Model

The following abstract data models used to represent provenance information were identified: *graphs* were the most frequent (18/44, 41%), followed by *lists* (12/44, 27%), *references* (eg, IDs or hash values; 3/44, 7%), combination of *graph and dictionary* (1/44, 2%), and *composite objects* (1/44, 2%). In total, 7% (3/44) of the publications did not specify the exact abstract data model used.

#### Concrete Data Model

The abstract data models described were implemented using the following concrete data models and associated storage solutions: *blockchain* (11/44, 23%) for list-based representations, *Resource Description Framework* (8/44, 18%) stored in triplestores for graph representations, and *relational model* (5/44, 11%) or *XML* (2/44, 5%) for different types of abstract models. Three solutions (3/44, 7%) used other file formats, such as binary or Hierarchical Data Format, Version 5 (HDF5) [71]. Many papers (7/44, 16%) did not provide specific information on the concrete data model used.

**Figure 5:**
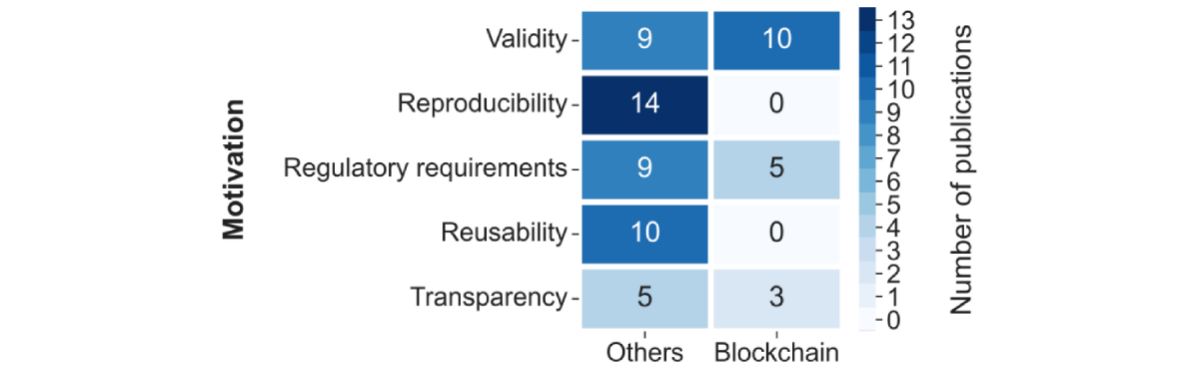
Frequency table for motivation groups and whether or not the solution is blockchain-based.

When cross-referencing the motivation categories versus whether the contribution was blockchain based or used some other technology (Figure 5), there is a clear picture showing that papers that described blockchain-based solutions did not refer to reproducibility or reusability. Given the immutable, transparent, and nonrepudiable nature of a blockchain, it is particularly well suited for applications focusing on validity or fulfilling regulatory requirements, which seems to be reflected in the motivation to implement this technology (refer to the *Principal Findings* section for further discussion).

#### Use of Provenance Standards

A total of 23% (10/44, 23%) papers stated compatibility with the PROV data model, whereas 7% (3/44, 7%) papers claimed compatibility with OPM. Most publications (31/44, 70%) did not state compatibility with either standard. Among all the papers that stated compatibility with either standard, all papers published since 2018 (7/44, 16%) preferred the PROV model. No paper mentioned compatibility with both standards.

#### Immutability

Data that cannot be altered once created or captured are considered immutable. Methods and solutions presented in 27% (12/44, 27%) publications provided immutability or nonrepudiability, of which 92% (11/12, 92%) were based on blockchain technology, which is inherently immutable. One paper stated nonrepudiable provenance based on cryptographic methods [61].

#### Materialization

We further analyzed whether the methods or solutions described store intermediate results as complete data sets, that is, *materialize* such data, or store only the metadata that led to these results, thus representing intermediate steps virtually. Most of the methods and solutions did not materialize intermediate results (31/44, 70%), whereas 20% (9/44, 20%) did. Interestingly, these papers described solutions focusing on omics (5/44, 11%) and imaging data (4/44, 9%), which makes sense, as processing and data generation are particularly expensive for these complex types of data (see also *Principal Findings* section).

### Functionality

#### Overview

**Figure 6:**
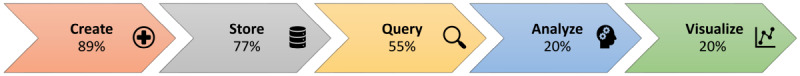
Steps of the data life cycle supported by the methods and solutions analyzed.

The technical activities supported by data provenance methods, models, and implementations are the creation or capture, storage, retrieval or query, analysis, and visualization of data provenance information, which are common activities in the data life cycle. When looking at the support provided for these activities by the methods and solutions analyzed, there was a clear decrease in support for tasks performed later in the data life cycle, as illustrated in Figure 6 (see also the *Principal Findings* section).

Several publications (39/44, 89%) described methods supporting multiple activities in the data life cycle. The frequency of support for individual steps was in ascending order: *create* (39/44, 89%; ie, all publications that contained information on support for this particular step of the life cycle), *store* (34/44, 77%), *query* (24/44, 55%), *visualize* (9/44, 20%), and *analyze* (9/44, 20%). Data storage has already been analyzed in the previous section. Therefore, in this section, we focus on a more detailed description of support for the remaining activities in the provenance data life cycle.

#### Creation or Capture

Among the papers that described methods or solutions supporting the creation or capture of provenance information (39/44, 89%), most papers (16/39, 41%) captured provenance information and metadata by changing a larger program, framework, or script used for data generation or processing to *additionally capture* the data needed. The second most common method for capturing provenance information, which is unique to blockchain-based solutions, was the *inherent capture* of provenance information using smart contracts (10/39, 26%). Some papers, including 1 using blockchain-based solution, described *integrated capture* solutions, such as a middleware- or trigger-based approaches that are transparent to applications or persistence layers (8/39, 21%), whereas others described methods based on provenance information from *external sources* such as research databases (6/39, 15%).

#### Querying or Retrieval

Among the papers that described methods or solutions supporting the querying or retrieval of provenance information (24/44, 55%), 25% (11/44) of papers relied on structured queries, using SQL, SPARQL, GraphQL, or similar query languages. A total of 42% (10/24) of solutions provided a graphical user interface or an application programming interface to retrieve the provenance metadata. Overall, 4% (1/24) of articles stated retrieval using unstructured queries, that is, a search string, and another (1/24, 4%) described a method using a selective query using unique identifiers. A total of 13% (3/24) of papers did not specify the method of retrieval.

#### Analysis

The support for the analysis of data provenance solutions can have many forms. In this study, the analyses were categorized as “generic” if they entailed generally applicable methods such as providing descriptive statistics and metrics as well as simple comparisons. Among the papers described methods or solutions supporting the analysis of provenance information (9/44, 20%), 44% (4/9) of papers fell into this category. Analyses were considered “specific” when they were tailored toward provenance-specific use cases, such as reasoning, validation tasks, and error tracing. A total of 44% (4/9) of papers fell into this category: 22% (2/9) of papers described ways to validate that data come from trustable devices [28,61], 11% (1/9) of papers described backward reasoning to identify problems regarding provenance in the data derived from monitoring systems [62], and 11% (1/9) of papers described the validation and identification of gaps in traceability in clinical research data [34]. Moreover, 11% (1/9) of additional articles described a range of approaches to analyzing provenance metadata, including *generic* and *specific* approaches [39].

#### Visualization

The results of generic analyses are typically visualized using common types of visualizations, such as bar and line charts. Among the papers that described methods or solutions supporting the visualization of provenance information (9/44, 20%), most papers (7/9, 78%) are based on some sort of graph- or flow network–based visualization. A total of 22% (2/9) of publications did not use such a basis but described methods or solutions showing digested information in bar charts and boxplots.

Visualization techniques or methods included dashboard-style combinations of multiple visualizations and metrics, Sankey diagrams, aggregations of graph nodes, force-directed graphs, tables, and an informal comic-style visualization of processes. Implementations are typically based on common visualization libraries or programs, such as D3.js, Gephi, yEd, sigma.js, Dagre, GraphViz, or Google Datalab.

#### Time of Generation

Of the solutions and methods capturing or creating provenance information (39/44, 89%), most (31/39, 79%) did so prospectively close to when the data were being processed. A minority (6/39, 15%) captured provenance information retrospectively after the processing concluded, based on the artifacts, such as log files, created. A total of 5% (2/39) of articles described the option for retrospective and prospective capture of provenance information, where one solution allows the reconstruction of provenance metadata for a previously finished process [46], and another captures provenance information from log files while also offering prospective capture via a plug-in functionality for workflow management systems [67].

## Discussion

### Outline

In this study, we provided an overview of the research on data provenance methods and technologies developed for or used in the biomedical domain. The methods and solutions described in the identified literature are heterogeneous. The supported functionalities and the design of methods were hence described to provide a systematization for navigating the heterogeneous landscape and to support the comparison of the functionalities and designs based on several characteristics. Furthermore, we identified gaps in the literature based on the systematization, including a lack of coverage regarding certain functionalities, such as the analysis of provenance metadata. The principal findings, related works, and limitations are presented in the following sections.

### Principal Findings

Despite the potential advantages of using data provenance technologies in biomedical research, as stated in the *Introduction* section (eg, improved reproducibility and data quality), and an increasing interest in the literature, as shown in the results on publication metadata, such technologies are still not widely adopted in the domain. The results of this scoping review reveal a heterogeneous landscape of methods, models, and implementations with very different objectives and, therefore, very different feature sets.

Regarding provenance aspects (where, how, why, and who), every solution analyzed in this review captures the aspect of *where* the data originates from. Where provenance is the core property of provenance and can be considered the most relevant aspect in the body of literature identified. The other aspects require more details to be included in provenance metadata but might not be required for all use cases and are, therefore, not supported in about half of the papers studied, possibly to reduce complexity. However, the answer to the question of how and why a data set was altered can be particularly important in biomedical research to ensure data reliability and auditability.

When looking at the logical and concrete data models used, graphs and graph databases are the most prevalent, which is reasonable, as these are natural representations for provenance information. Widespread generic data models, such as the relational model or XML, are also used frequently, as they are versatile enough to support provenance metadata from a wide range of implementations. Although some approaches already adopted or are at least compatible with the most common provenance standards, PROV and OPM, many papers did not address compatibility with standards, which hinders the interoperability of provenance metadata.

The PROV model has gained popularity in the recent years. It is slightly newer and more comprehensive than OPM, which was the “first community-driven model for provenance” [72]. PROV is more mature and consists of several documents describing concepts, notations, ontologies, and interoperability options, for example, with existing metadata standards, such as the Dublin Core [8,9]. Furthermore, PROV allows for more detailed modeling of relations regarding entities or agents [73]. Consistent support of the PROV model could foster the compatibility of solutions and widen the areas of application. For instance, provenance metadata gathered by one solution can be analyzed using an entirely different solution, as long as both solutions are PROV compatible.

The processing or generation of large and complex data, such as omics or images, is costly [74], and in many cases, it may not be feasible to repeat the whole process, if needed. Storing a complete data set of intermittent results helps save time and resources, should the processing procedure change and be replicated or should other processing paths be explored. Hence, the materialization of intermediate processing steps is often implemented in pipelines for such purposes. Furthermore, the methods in the respective articles addressing such types of data are often motivated by reproducibility aspects, which may be attributed to the processing complexity and sheer volume of the data, which increases the difficulty of reproducing the results and the processing itself.

Recently, blockchains have established themselves as a technology that supports some aspects of data provenance. Blockchains inherently provide where provenance and immutability by facilitating consensus algorithms and cryptographic methods to maintain a single list of blocks, where all involved parties agree on a predecessor and successor for any given block. These blocks usually contain transaction information, thus enabling where provenance for the data included or referenced. Unfortunately, the blockchain-based solutions we identified and analyzed in this review often do not go beyond their inherent property and, at this stage, provide little coverage for other aspects, such as reproducibility and reusability, which are much needed in biomedical research. However, because of their support for clearly defined and immutable lineage, they can be well suited for meeting regulatory requirements (eg, providing audit trails).

The creation or capture of data provenance information is logically the first step toward its use. Therefore, it is not surprising that creation and capturing is the most commonly supported activity in the provenance data life cycle in all the methods or solutions analyzed. Provenance data analysis and visualization were addressed less frequently, which could be a direct result of the fact that data provenance is still underutilized in biomedical research, and hence methods for the “use” of provenance information are developed or studied more rarely. We believe that the development of domain-specific analysis and visualization methods could be an important step to practically demonstrate the added value of provenance tracking and help increase its adoption. Furthermore, we did not find any indication of reference data sets, which could be used to develop and evaluate analysis or visualization methods for provenance data.

Finally, we found that most solutions or methods analyzed rely on methods for additionally capturing provenance data, whereas only a small number of approaches rely on integrated capture methods that are transparent to the user or the processing environment. This implies that there is quite a lot of effort involved in capturing provenance data information, which may point toward an additional field of promising research on how to transparently capture provenance information without causing additional work on the side of the users or developers of data processing frameworks.

### Related Work

Several related papers have studied and systematized research on data provenance, albeit typically with a focus on general concepts or applications and not on biomedicine. In 2005, Simmhan et al [6] introduced a taxonomy of properties of provenance technologies, which shows some similarities with the data items defined for this review, such as the specific use of provenance (cf *motivation*), the method for provenance dissemination (cf *retrieval or querying*), and the provenance representation used (cf *data models*). In addition, the authors focused on more technical properties, such as the granularity and level of detail of the provenance metadata used and the scalability or storage overhead of their management.

A more recent (2017) survey by Herschel et al [3] states that the definition of provenance can be interpreted in different ways, resulting from different applications and technical requirements, and gives an overview of the research field. Although the survey is not limited to the biomedical context, the wide spectrum and heterogeneity of motivations (ie, applications and technical requirements) to use provenance can also be seen in our results. This includes the applications of provenance, the memory footprint and interoperability, query expressiveness, application integration, and the data provenance of existing results (cf *motivation*, *data models, functionality, and provenance aspects).* One central challenge identified by the authors is the need for more research on the analysis and visualization of provenance data: “While querying provenance data has been studied together with data models for provenance, there exists only little work on properly visualizing, exploring, and analyzing provenance data in a user-friendly way” [3]. Considering our results, this is also the case for provenance in the biomedical context (cf *functionality*).

de Lusignan et al [4] reviewed research using routine clinical data to identify key concepts of data readiness, which also included data quality and provenance. One of their conclusions is that the description of metadata should be formalized to benefit “the validity of research findings based on routinely collected data” in the context of health care and health care informatics. The authors further introduced a distinction between primary and secondary data provenance: primary provenance refers to the origin of the data (ie, without the knowledge of processing applied until that point), and secondary data provenance refers to the processing done after retrieving the original data [4]. In our work, these are called *Where provenance* and *How,* and *Why* provenance, respectively.

Goble [75] provided an informal yet comprehensive discussion and outline of provenance. The paper covers aspects of provenance as “the 7 W’s (Who, What, Where, Why, When, Which, (W)how),” which remain unspecified, as well as general use cases or motivations for facilitating provenance. Furthermore, it phases the question of whether provenance metadata are intrinsically immutable, which we investigated from a technical perspective in this review (cf *immutability*). The paper also discusses provenance data models and how provenance metadata should accompany the data they describe along the processing path, which is described by *provenance aspects* and data *representation and storage* in this review.

Last year, Gierend et al [76] published a protocol for a scoping review on biomedical data provenance. The actual review has not been published yet. According to the RQs stated and data items listed, the review will focus on use cases and aspects such as the value and usability of provenance information (RQ 2), challenges and problems encountered (RQ 3), guidelines and requirements for provenance in the biomedical domain (RQ 4), and issues around the completeness of provenance information (RQ 5). By contrast, our review has a stronger focus on systematizing and comparing the technical aspects of provenance data management in biomedicine. This partly overlaps with the first RQ posed by Gierend et al [76], which is to identify approaches for the classification and tracking of provenance criteria. However, it is highly likely that our analysis provides more depth, as we focused specifically on methodological and technical perspectives, which is, for example, also reflected by including the IEEE Xplore database in our search.

### Limitations

This study has some limitations owing to the chosen search strategy, heterogeneity of the discovered and included articles, and methods and solutions described therein. Most importantly, the search strategy was designed to specifically capture the topic of provenance in biomedical research, and the terms used did not explicitly include specific research domains, such as psychology or other behavioral sciences. However, we believe that our literature selection strategy likely only missed relevant articles that did not address the broader context in their abstracts, which meant mentioning one of the keywords used in our search process. Furthermore, we consider the existence of a large body of literature with these characteristics unlikely. The fact that approximately 46% (44/96) of all identified unique references were included in this review can be taken as an indicator that provenance tracking has not yet become a common feature of biomedical research platforms. If this were the case, it would be expected that a greater proportion of the literature would have mentioned provenance as a sidenote, leading to its exclusion owing to a lack of focus on provenance technology. By contrast, many articles mentioning provenance in their titles or abstracts have a specific focus on this topic.

The methods and solutions described in the selected articles were systematized, important properties were qualitatively identified, their occurrences were assessed and reported, and individual examples were included for special cases that appeared rather unique. The reported statistics are subject to uncertainties. They should be understood as indications and do not describe the entire field with absolute certainty.

### Conclusions

Despite the growing interest in the literature, little progress has been made in the biomedical field regarding the development of data provenance technologies, which could help mitigate reproducibility issues. An important reason could be a lack of generic and transparent solutions for easily capturing or creating provenance data, resulting in potentially substantial efforts for provenance tracking. Another gap we identified is a lack of specific methods for analyzing and visualizing provenance data, which may make it difficult to adequately leverage the added value provided. We also observed quite some heterogeneity in the motivation, scope, and functionality of provenance tracking methods for biomedical applications, pointing toward a potential lack of a unified understanding of underlying concepts and a narrow focus on specific use cases. Providing general purpose data sets and application scenarios, as well as benchmarking mechanisms, could help overcome this challenge in the future.

Our work focused specifically on papers from the biomedical field to investigate the state of the art in this particular application area. In future work, it may be worthwhile to also study general purpose methods, models, and implementations and investigate their applicability to biomedical use cases.

## Appendix

Multimedia Appendix 1 Queries used for the database search.

Multimedia Appendix 2 Selection process and collected data.

## Abbreviations

HDF5: Hierarchical Data Format, Version 5
OPM: Open Provenance Model
PRISMA-ScR: Preferred Reporting Items for Systematic Reviews and Meta-Analyses Extension for Scoping Reviews
RQ: research question
W3C: World Wide Web Consortium
