# Supplementary material for: Data Provenance in Biomedical Research: Scoping Review
Source: J Med Internet Res. 2023 Mar 27;25:e42289. doi: 10.2196/42289 (PMC10132013; doi:10.2196/42289)
Supplement: Multimedia Appendix 1 [file jmir_v25i1e42289_app1.docx]

# Appendix A: Queries used for database search

The searches were performed on 07.02.2022 from a computer within the network of Charité – Universitätsmedizin Berlin in Germany.

## PubMed

(("data provenance"[Title/Abstract]) OR ("data lineage"[Title/Abstract]) OR ("data pedigree"[Title/Abstract])) AND ((medical[Title/Abstract]) OR (biomedical[Title/Abstract]) OR (health[Title/Abstract])) AND (("2010"[Date - Publication] : "2021"[Date - Publication]))

## IEEE Xplore

(("All Metadata":"data provenance") OR ("All Metadata":"data lineage") OR ("All Metadata":"data pedigree")) AND (("All Metadata":medical) OR ("All Metadata":biomedical) OR ("All Metadata":health))

## Web of Science

(TS=("data provenance") OR TS=("data lineage") OR TS=("data pedigree")) AND (TS=(medical) OR TS=(biomedical) OR TS=(health))
